# Periostin Associates with Notch1 Precursor to Maintain Notch1 Expression under a Stress Condition in Mouse Cells

**DOI:** 10.1371/journal.pone.0012234

**Published:** 2010-08-18

**Authors:** Hideyuki Tanabe, Issei Takayama, Takashi Nishiyama, Masashi Shimazaki, Isao Kii, Minqi Li, Norio Amizuka, Ken-ichi Katsube, Akira Kudo

**Affiliations:** 1 Department of Biological Information, Tokyo Institute of Technology, Yokohama, Japan; 2 Division of Oral Health Science, Department of Developmental Biology of Hard Tissue, Graduate School of Dental Medicine, Hokkaido University, Sapporo, Japan; 3 Department of Molecular Pathology, Graduate School of Tokyo Medical and Dental University, Tokyo, Japan; Universität Heidelberg, Germany

## Abstract

**Background:**

Matricellular proteins, including periostin, modulate cell-matrix interactions and cell functions by acting outside of cells.

**Methods and Findings:**

In this study, however, we reported that periostin physically associates with the Notch1 precursor at its EGF repeats in the inside of cells. Moreover, by using the periodontal ligament of molar from periostin-deficient adult mice (Pn−/− molar PDL), which is a constitutively mechanically stressed tissue, we found that periostin maintained the site-1 cleaved 120-kDa transmembrane domain of Notch1 (N1™) level without regulating Notch1 mRNA expression. N1™ maintenance *in vitro* was also observed under such a stress condition as heat and H_2_O_2_ treatment in periostin overexpressed cells. Furthermore, we found that the expression of a downstream effector of Notch signaling, Bcl-xL was decreased in the Pn−/− molar PDL, and in the molar movement, cell death was enhanced in the pressure side of Pn−/− molar PDL.

**Conclusion:**

These results suggest the possibility that periostin inhibits cell death through up-regulation of Bcl-xL expression by maintaining the Notch1 protein level under the stress condition, which is caused by its physical association with the Notch1 precursor.

## Introduction

Cells interact with the extracellular matrix (ECM) and its associated molecules such as growth factors, cytokines, and proteases that modulate cells. These interactions are modulated by matricellular proteins, components of the ECM including thrombospondins, secreted protein acidic and rich in cysteine (SPARC/osteonectin), osteopontin, tenascins, and the CCN family of proteins, which have characteristic expression patterns and are highly expressed during development and in remodeling and repairing of wound tissues of adult mice [1]. These components do not seem to have direct structural roles but modulate cell functions such as collagen fibrillogenesis, cell adhesion, migration, proliferation, and differentiation by their binding to various molecules; such as cell-surface receptors, growth factors, cytokines, proteases, and type I collagen [1].

Periostin, a 90-kDa secreted protein, which is a member of the fasciclin I family [2], [3], has been recently recognized as a matricellular protein [4]. Expression of periostin is principally observed in mesenchymal tissues during embryogenesis, and, in the adult, it is restricted to certain tissues such as periodontal ligament, periosteum [2], and cardiac valves [5], which are mechanically stressed tissues, and healing tissues [6], [7], [8], [9]. Periostin, therefore, is expected to play an important role under the stress condition; indeed, it is reported that periostin maintains the integrity of the periodontal ligament (PDL) during occlusal loading in mice [10]. Regarding the molecular function of periostin, it associates with the ECM components such as collagen I [11] and integrins, which supports cell adhesion and migration [9], [12].

For further analysis of periostin function, periostin-deficient (Pn−/−) mice have been generated and well studied. These mice show the disappearance of the shear zone in the incisor PDL [13], a reduced diameter of collagen fibrils in the skin [11], and a high occurrence of rupture after myocardiac infarction [8], [9]. These abnormalities are principally attributed to the defects in collagen fibrillogenesis. Furthermore, it was also shown that the integrin-mediated FAK signaling is reduced in myocardial infarcts of Pn−/− mice [9], suggesting reduced cell motility in Pn−/− cells. Because of these findings, periostin is recognized as an important player in the modulation of collagen fibrillogenesis and integrin signaling. However, considering that periostin is expected to be a multifunctional protein, collagen fibrillogenesis or integrin signaling might not be the only processes in which periostin is involved. Recent studies have indicated that matricellular proteins regulate the Notch signaling pathway [14], [15]. Notch signaling plays a central role both in development and in adult tissue homeostasis that regulates a variety of cell functions such as cell migration, proliferation, differentiation, and apoptosis. This signaling is activated by ligands such as Delta-like and Jagged, which bind to mature cell-surface Notch receptors (Notch1,2,3,4), resulting in the activation of transcriptional factors such as Hes and Hey family proteins [16].

In this study, we investigated whether periostin influences Notch signaling. The results demonstrated that periostin physically associated with the Notch1 precursor to maintain its protein expression level and affected the subsequent Notch signaling under a stress condition.

## Results and Discussion

### Periostin physically associates with Notch1 precursor at its EGF repeats

**Figure 1:**
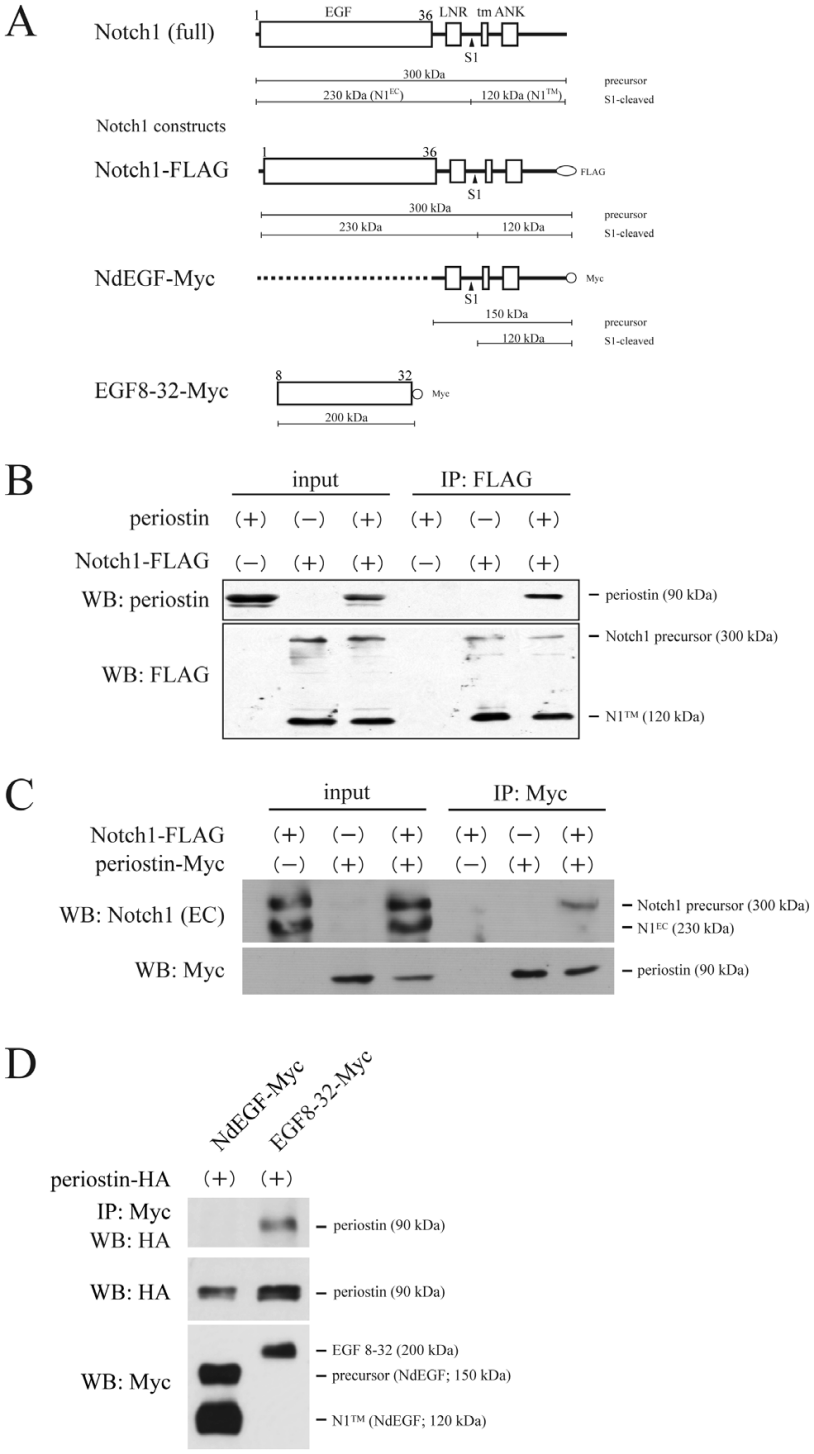
Periostin associates with Notch1 precursor in its EGF repeats. (A) Schematic illustration of Notch1 and the Notch1 expression constructs used in this study. EGF, epidermal growth factor-like repeats; LNR, Lin-12/Notch repeats; tm, transmembrane region; ANK, Ankyrin repeats; S1, site 1 (S1) cleavage point. Notch1 precursor (300 kDa), following the glycosylation step, is cleaved at the S1 site and forms a heterodimeric protein consisting of the extracellular domain (N1^EC^; 230 kDa) and the transmembrane domain (N1™; 120 kDa). All the expression constructs were tagged with FLAG or Myc in their C-terminus. NdEGF-Myc lacks EGF repeats of Notch1, and EGF8-32-Myc consists of the 8th-32nd of EGF motifs. Molecular weights of these constructs are indicated in the scheme. (B) Co-immunoprecipitation of periostin along with Notch1. Whole-cell lysates of HEK293T cells expressing the indicated proteins were immunoprecipitated with anti-FLAG antibody, and were then Western-blotted with anti-periostin antibody (top panel; periostin, 90 kDa) or anti-FLAG antibody. Anti-FLAG antibody recognized the Notch1-FLAG precursor (300 kDa) and N1™-FLAG (120 kDa) (bottom panel). The left 3 lanes show input products; and the right 3 lanes, IP products. (C) Co-immunoprecipitation of Notch1 along with periostin. Whole-cell lysates of HEK293T cells expressing the indicated proteins were immunoprecipitated with anti-Myc antibody and then Western-blotted with anti-Notch1 (recognizing extracellular region, EC) antibody or anti-Myc antibody. Anti-Notch1 (EC) antibody recognized the Notch1-FLAG precursor and N1^EC^ (230 kDa), (top panel), and anti-Myc antibody recognized periostin-Myc (bottom panel). (D) Co-immunoprecipitation of the deletion constructs of Notch1 with periostin. Whole- cell lysates of HEK293T cells expressing the indicated proteins were immunoprecipitated with anti-Myc antibody and subsequently Western-blotted with anti-HA antibody. Anti-HA antibody recognized periostin-HA (top panel). Bottom 2 panels show input products: the upper one shows periostin-HA; and the lower one, NdEGF-Myc, the precursor (150 kDa) and N1™-Myc in the left and EGF8-32-Myc (200 kDa) in the right.

To investigate the effect of periostin on Notch signaling, we first examined whether periostin could physically associate with Notch1; because matricellular proteins often show their function through protein-protein interactions with cell-surface proteins [1]. For this experiment we prepared several Notch1 and periostin constructs to transfect HEK293T cells. It was previously reported that Notch1 is first synthesized as a 300-kDa precursor protein and that following the glycosylation step, Notch1 is cleaved by a furin-like convertase (site 1 cleavage; S1 cleavage) to form a heterodimer receptor composed of a 230-kDa extracellular domain (N1^EC^) and a 120-kDa transmembrane domain (N1™); and then the mature heterodimeric Notch1 is trafficked to the cell surface [17], [18]. The various Notch1 protein structures and related tagged Notch1 constructs used in this experiment are shown in Fig. 1A. Firstly, we transfected HEK293T cells with Notch1-FLAG and periostin constructs and then examined their expression by Western blot analysis using an anti-FLAG or anti-periostin antibody. Whole-cell lysates from the transfectants revealed 2 bands of Notch1, the precursor and the N1™ of the S1-cleaved heterodimeric Notch1 and the periostin (Fig. 1B). To detect the association of Notch1 and periostin proteins, we performed immunoprecipitation of Notch1 by using anti-FLAG antibody followed by Western blot analysis using anti-periostin antibody, revealing the presence of periostin in the precipitate of Notch1 (Fig. 1B). Next, we performed the reverse experiment, as shown in Fig. 1C. We immunoprecipitated periostin using an anti-Myc antibody from the another whole-cell lysates of HEK293T transfectants that co-expressed Notch1-FLAG and periostin-Myc constructs and then performed Western blot analysis using an antibody raised against the extracellular region of rodent Notch1. The data revealed the presence of the Notch1 precursor in the immunoprecipitate; however, S1-cleaved N1^EC^ of heterodimeric Notch1 failed to be detected in the precipitate, indicating that periostin physically associated with the Notch1 precursor.

Next, we determined the domain of Notch1 that associated with periostin. Notch1 is composed of 36 EGF-like motifs (EGF) and Lin-12/Notch repeats (LNR) in its N1^EC^, a transmembrane domain (tm), Ankyrin repeats (ANK), and other motifs including the nucleus localization signal in N1™ [19], as shown in Fig. 1A (top figure). To find this association domain, we performed another transfection experiment using a C-terminus Myc-tagged deletion construct of Notch1 lacking the EGF motifs (NdEGF-Myc) and a construct with 8th-32nd EGF motifs (EGF8-32-Myc) together with the periostin-HA construct; and then immunoprecipitation was performed using anti-Myc antibody (Fig. 1D). In the result, periostin was co-immunoprecipitated with EGF8-32, whereas it failed to be detected with NdEGF consisting of two different forms of NdEGF, i.e., the precursor form and the S1-cleaved heterodimeric form, indicating that periostin physically associated with the EGF repeats of Notch1. Since periostin was reported to be a matricellular protein [4], periostin is believed to play a role at the cell surface. Indeed, a recombinant periostin, when present in the culture medium was reported to activate integrin signaling [9]. However, our results suggest another role for periostin, i.e., association with EGF motifs of the Notch1 precursor inside the cell during membrane trafficking.

### 120-kDa transmembrane domain of Notch1 (N1™) is decreased in Pn−/− molar PDL

**Figure 2:**
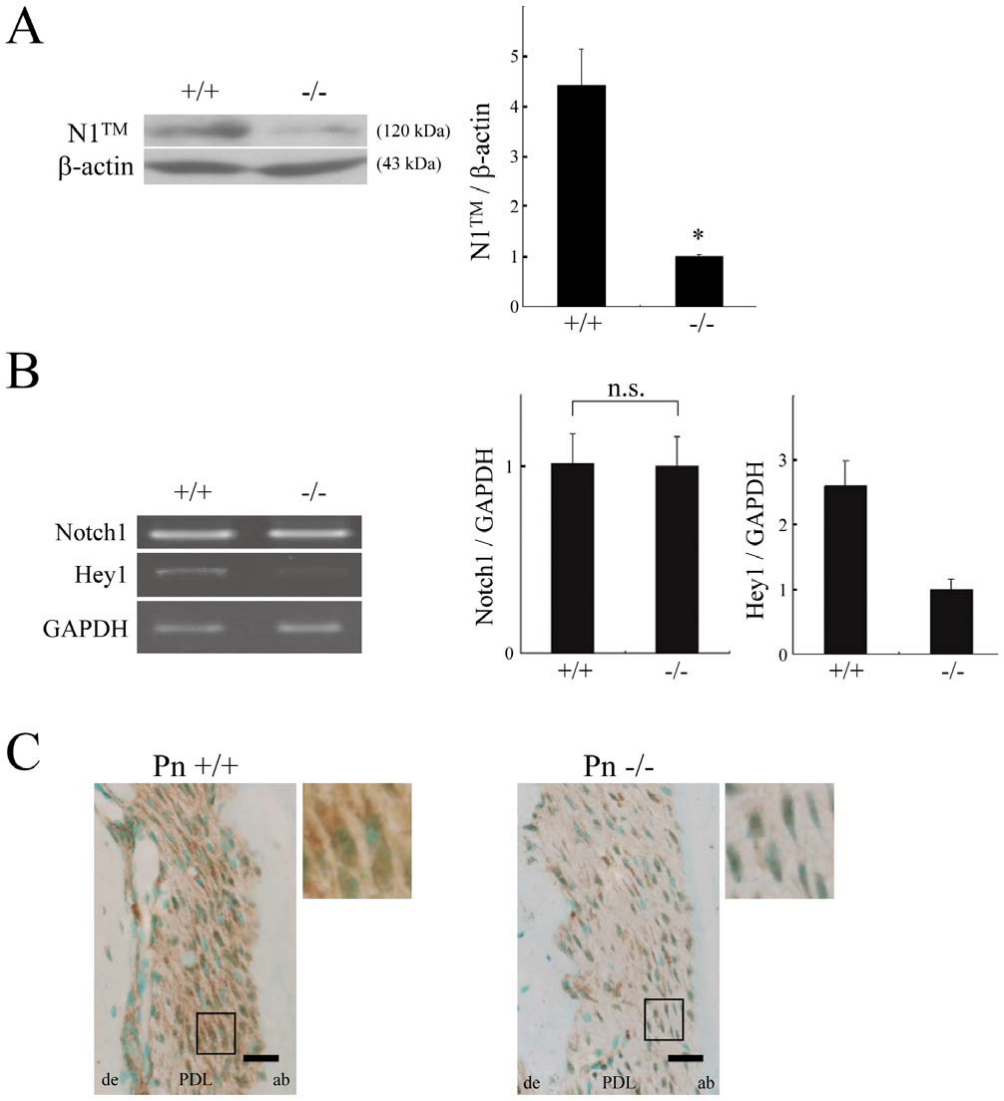
120-kDa Notch1 transmembrane domain (N1™) is decreased in Pn−/− molar PDL. (A) Western blot analysis of molar PDL from a 12-week-old mouse, with anti-Notch1 antibody for detection. β-actin was used for a loading control. Results indicate the relative density normalized to the β-actin. (means±SEM, n = 4; *p<0.05.). (B) Semi-quantitative RT-PCR for Notch1 and Hey1 mRNAs in molar PDL from 12-week-old mice. GAPDH was used for a loading control. Results indicate the relative density normalized to the GAPDH. (means±SEM, n = 3; n.s.; not significant difference, p = 0.06 for Hey1). (C) Immunostaining of Notch1 in PDL of molar teeth from 12-week-old Pn+/+ and Pn −/− mice. The nuclei were counter stained with methyl green. Boxed areas are enlarged at the top right of each panel. de, dentin; PDL, periodontal ligament; ab, alveolar bone. Bars, 50 µm.

To investigate whether periostin affects Notch signaling *in vivo*, we employed PDL of mandibular molars from adult mice. We first examined the Notch1 expression level by Western blot analysis using antibody against the C-terminal part of Notch1. In the result, although we could not detect Notch1 precursor, we did detect the 120-kDa N1™ band of S1-cleaved heterodimeric Notch1, indicating that Notch1 was expressed in the molar PDL (Fig. 2A). We also examined Notch1 expression in the Pn−/− molar PDL and found that the N1™ level was decreased to 25% of the level observed in the wild-type molar PDL (Fig. 2A), suggesting that S1-cleaved heterodimeric Notch1 was decreased in Pn−/− molar PDL. To confirm whether this decrease was a reflection of reduced transcription, we performed semi-quantitative RT-PCR analysis for Notch1, but observed no significant difference in the transcription level (Fig. 2B). This result suggests that periostin affected Notch1 expression in post-transcriptional manner. Furthermore, we also performed the semi-quantitative RT-PCR analysis for Hey1, a downstream effector of Notch signaling, and found that its expression level was decreased to 40% in the Pn−/− molar PDL compared with that in the wild-type one (Fig. 2B), suggesting that Notch signaling was decreased in the Pn−/− molar PDL, consistent with the decrease of N1™ in the Pn−/− molar PDL shown in Fig. 2A. Next, we performed immunohistochemical analysis of Notch1 in the mandibular molar (Fig. 2C). In the wild-type molar PDL, we observed broad areas immunopositive for Notch1, indicating that Notch1 was expressed in molar PDL. On the other hand, in the Pn−/− molar PDL, Notch1 staining was apparently decreased, however, it was detectable intracellularly, indicating that the localization of mature Notch1 at the cell-surface was reduced and the immature Notch1 remained inside of the cells in the Pn−/− molar PDL. This observation suggests that Notch1 proteins were synthesized in the Pn−/− molar PDL but that their maturation had been disturbed and that many of the Notch1 proteins might have been degraded before they were transported to the cell surface.

Reduced localization of mature Notch1 at the cell-surface was also observed in the periosteal cells of the femur of 12-week-old Pn−/− mice compared with that in the wild-type ones (Fig. S1), suggesting that periostin might regulate Notch1 expression in various tissues that express periostin.

Taken together, the above data led us to propose that periostin affected Notch1 maturation by physical association with its precursor inside of the cells to increase the localization of mature Notch1 protein at the cell surface.

Although this physical association of periostin with the Notch1 precursor might be appropriate, by the Western blot analysis, we detected the N1™ of heterodimeric Notch1 in the wild-type molar PDL, whereas we could not detect its precursor. It is possible that a properly folded Notch1 precursor is rapidly S1-cleaved and then transported to the cell surface, with the result being that the precursor form does not remains at a detectable level by Western blot analysis.

Recently, the possibility of an intracellular function of matricellular proteins was proposed, since (1) SPARC is located primarily intracellularly in non-pathological tissues, and is coincidently located with protein disulfide isomerase (PDI), a ER-resident protein, in pseudoachondroplasia chondrocytes [20], and (2) periostin appeared to be anchored in the Golgi [21]. In light of these previous studies, we can hypothesize an intracellular function of matricellular proteins active in the membrane trafficking pathway.

### Periostin maintains N1™ under a stress condition

**Figure 3:**
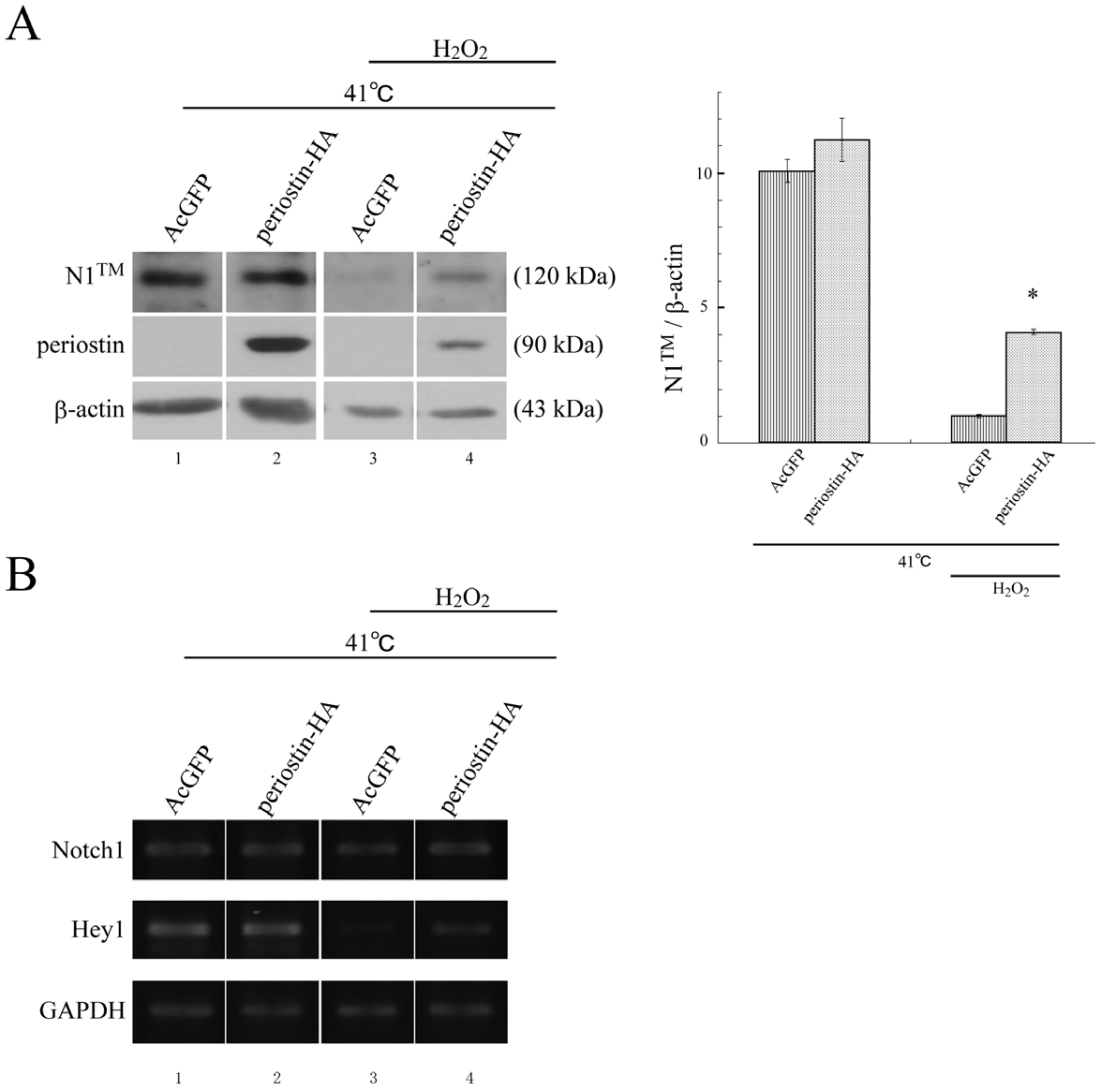
Reduction in Notch1 expression is suppressed in periostin-HA ST2 infectants. ST2 cells were retrovirally infected with the expression constructs of periostin-HA or AcGFP. Confluent ST2 cells were cultured for 1 week, and then the temperature condition of all the cells was shifted from 37°C to 41°C without or with 200 µM H_2_O_2_. Cells were cultured under this stress condition for 6 days with 1% FBS. After that, cells were collected for Western blot analysis or semi-quantitative RT-PCR analysis. (A) Western blot analysis for Notch1 of retrovirally infected periostin-HA ST2 cells. β-actin was used for a loading control. The right 2 lanes show the heat + H_2_O_2_-treated samples; and the left 2 lanes, heat treatment only. Results indicate relative density normalized to theβ-actin. (meansS±EM, n = 3; *p<0.05.). (B) Semi-quantitative RT-PCR analysis for Notch1 and Hey1. GAPDH was used for a loading control. The right 2 lanes show the heat + H_2_O_2_-treated samples; and the left 2 lanes, those with heat treatment only. The Notch1 mRNA level was not significantly changed, but Hey1 mRNA expression was increased in retrovirally infected periostin-HA ST2 cells compared with that in the AcGFP ones. The representative data from 3 independent experiments is shown.

To further assess the effect of periostin on Notch1 expression, we established retroviral infectants of a bone marrow-derived stromal cell line, ST2, by using the periostin-HA construct and an AcGFP construct for a control. Firstly, we performed Western blot analysis for detecting endogenous Notch1. Contrary to our expectation, the N1™ level was not significantly different between periostin-HA and AcGFP cells (Fig. S2A, cf., left 2 lanes). Since the PDL is always exposed to mechanical stress during mastication, we exposed the ST2 infectants to stress to mimic the circumstance of the PDL. For exposure to stress, we employed heat or heat and H_2_O_2_ treatment, which are known to induce reactive oxygen species (Ros); and a high level of Ros induces oxidative stress, as does like mechanical stretch [22]. Six days after the shift of the temperature condition of all cells from 37°C to 41°C without or with 200 µM H_2_O_2_ treatment, we performed Western blot analysis for Notch1 protein (Fig. 3A), and the semi-quantitative RT-PCR analysis for Notch1 mRNA (Fig. 3B). The N1™ protein level and the Notch1 mRNA level were not significantly different between periostin-HA cells and AcGFP cells under the non H_2_O_2_ treatment condition (Fig. 3A, B, cf., lanes 1, 2); however, in the heat+H_2_O_2_ treatment condition, the N1™ level was significantly deceased in the control AcGFP cells (Fig. 3A, cf., lanes 1, 3); whereas only a slight decrease was observed in the Notch1 mRNA level (Fig. 3B, cf., lanes 1, 3), suggesting that the S1-cleved heterodimeric Notch1 is difficult to be maintained under this stress condition. In this case, the N1™ level in periostin-HA cells was significantly higher than that in the AcGFP cells (Fig. 3A, cf., lanes 3, 4), whereas no significant difference was observed in the Notch1 mRNA level (Fig. 3B, cf., lanes 3, 4), suggesting that periostin maintained the N1™ level under the stress condition. The maintenance of N1™ level was also observed at a higher concentration of H_2_O_2_ (2.4 mM) treated periostin-HA cells (Fig. S2A). These data suggest that H_2_O_2_ induces the defect in Notch1 maturation, but periostin suppresses the H_2_O_2_ action, and maintains the N1™ level under the H_2_O_2_-induced oxidative stress condition, similarly to Fig. 3A. Furthermore, we also performed semi-quantitative RT-PCR analysis for Hey1 under the heat+H_2_O_2_ condition. Hey1 expression in the AcGFP cells was decreased by the heat+H_2_O_2_ treatment (Fig. 3B, cf., lanes 1, 3); however, it was maintained in the periostin-HA cells (Fig. 3B, cf., lanes 3, 4). This result supports the periostin function for maintenance of Notch1 protein shown in Fig. 3A, suggesting that periostin maintains both Notch1 protein expression and subsequent signaling under the stress condition.

The molecular system of how periostin maintained the N1™ level was not defined here. However, taken the next previous reports; ER resident proteins were the predominant targets of the H_2_O_2_-induced oxidative stress [23], and protein folding in ER became defective under the H_2_O_2_-induced oxidative stress condition [24], a possible explanation for this finding is that periostin supports protein folding of Notch1 precursor in the ER under the stress condition. In this scenario, without periostin, Notch1 precursor can not be well folded under the stress condition, causing the insufficient S1-cleaved heterodimeric Notch1 that is probably easily degraded, resulting in reduction of the N1™ level.

These *in vitro* data are consistent with the data on the N1™ level in the molar PDL (Fig. 2A), suggesting that this *in vitro* system has some similarities to the tissue-environment of the PDL and that mechanical stretch-induced oxidative stress might negatively influence Notch1 maturation.

It has been reported that a mutation in the EGF motifs of Notch3, which would induce misfolding in these motifs, causes a reduction in the cell-surface level of Notch3 in the Cerebral Autosomal Dominant Arteriopathy with Subcortical Infarcts and Leukoencephalopathy (CADASIL) mutant [25], suggesting the importance of the maturation event of Notch proteins for their cell-surface expression, coincident with our hypothesis about Notch1 protein expression.

### Bcl-xL expression is decreased in Pn−/− molar PDL cells

**Figure 4:**
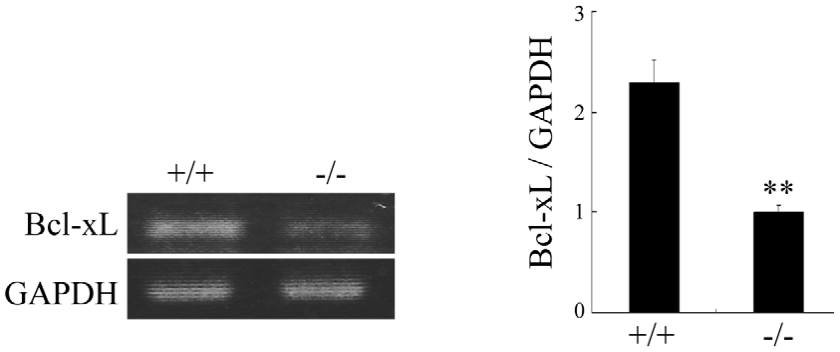
Bcl-xL expression is decreased in Pn−/− molar PDL. Semi-quantitative RT-PCR analysis of molar PDL from 12-week-old wild-type and Pn−/− mice. Bcl-xL expression was decreased in Pn−/− molar PDL. GAPDH was used for a loading control. Results indicate the relative density normalized to the GAPDH. (means±SEM, n = 3; **p<0.01.)

We next investigated whether the reduction in mature Notch1 level would affect survival of PDL cells *in vivo*. Since a cyclically induced strain, one of mechanical stress, reduces the expression of the cytoprotective factor Bcl-xL in a Notch signaling-dependent manner, and ultimately induces cell death in vascular smooth muscle cells *in vitro* [26], we focused on Bcl-xL expression. When we performed semi-quantitative RT-PCR analysis for Bcl-xL expression, its level in the Pn−/− molar PDL was decreased to 45% of that in the wild-type one (Fig. 4), consistently with the decrease of N1™ as shown in Fig. 2A, suggesting that periostin controls Bcl-xL expression, probably in a Notch signaling-dependent manner. Furthermore, we also demonstrated the maintenance of Bcl-xL expression in the periostin-HA cells under 2.4 mM H_2_O_2_ treated condition (Fig. S2A), and that this maintained Bcl-xL expression was reduced by a Notch signaling inhibitor: γ-Secretase inhibitor (Fig. S2B), suggesting that periostin controls Bcl-xL expression through the regulation of Notch signaling under the H_2_O_2_-induced oxidative stress condition. These data are consistent with the decrease of Bcl-xL expression in Pn−/− molar PDL, shown in Fig. 4.

### Stress tolerance is reduced in Pn−/− molar PDL

**Figure 5:**
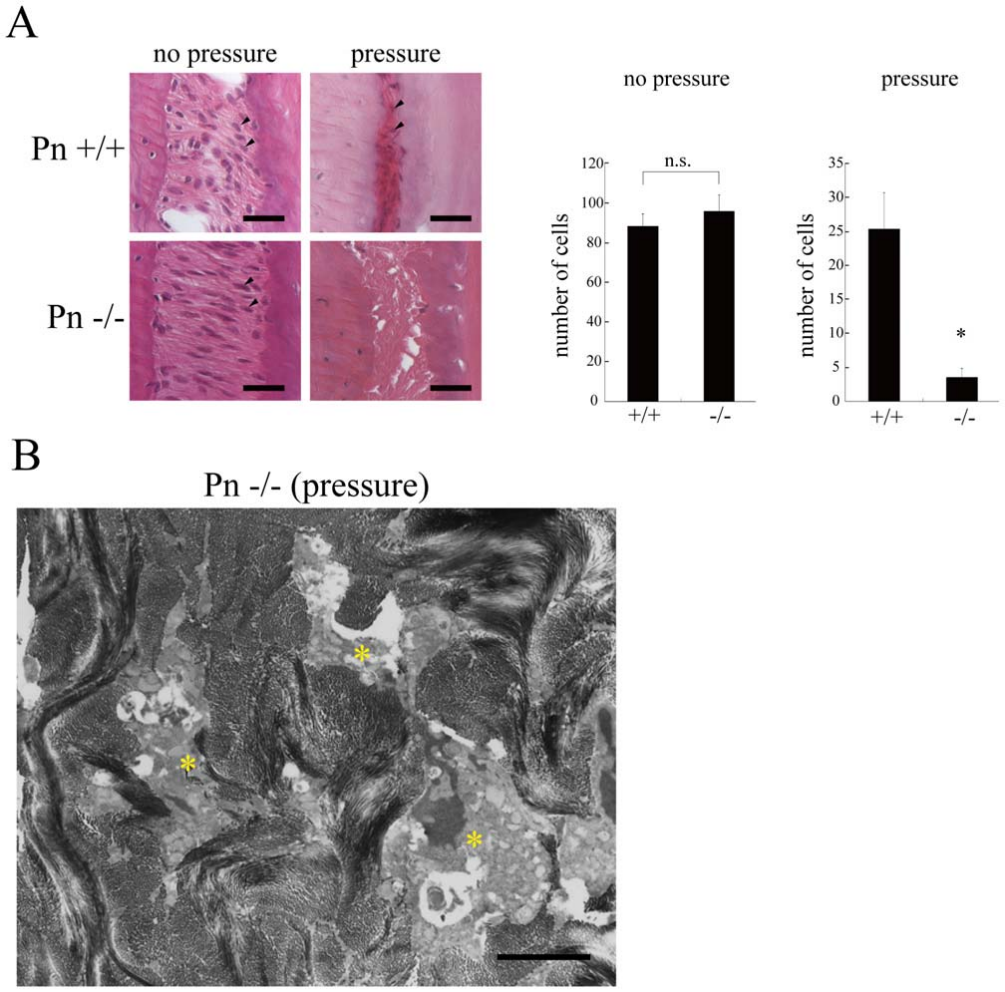
Living cells are decreased in the PDL on the pressure side of a Pn−/− molar tooth subjected to experimental tooth movement. Experimental model for tooth movement: The molars of 12-week-old mice were used for the experiments. (A) H-E-stained pressure side of the PDL. The tissues were markedly compressed by the elastic force. Cells, in which the nuclei were stained with hematoxylin, were determined as living cells. There were rarely observed in molar Pn−/− PDL, although they were apparent in the wild-type, as shown by the arrowheads. Bars, 50 µm. The cell number in the periodontal ligaments (1 mm distant toward apical and coronal sides from the center of the height of interradicular alveolar septum) on pressure (compression) and non-pressure (tensile) side of both wild type and Pn−/− were counted as living cells. (mean±SEM, n = 6; n.s.; not significant difference, * P<0.05). (B) Electron microscopic analysis of the pressure side of a Pn−/− molar PDL 3 days after the experimental tooth movement. Dead periodontal fibroblasts (asterisks) revealed vacuoles of various size, pyknotic nuclei, and collapsed cell bodies. Bars, 10 µm.

The decreased Bcl-xL expression in the Pn−/− molar PDL suggests that the tolerance against stress would be reduced, and possibly cell death will be enhanced in a high stress condition. To further demonstrate the reduced tolerance against stress in the Pn−/− molar PDL, we next performed the experimental molar tooth movement, which induces a strong stress on the molar PDL. Most of cells showed cell death at the pressure side of the Pn−/− molar PDL, although we observed apparently living cells at that side of the wild-type (Fig. 5A, B), suggesting that cells in the Pn−/− molar PDL could not tolerate stress compared with those in the wild-type molar PDL. These data are consistent with those in Fig. 4 showing decreased Bcl-xL expression in the Pn−/− molar PDL. Furthermore, cell viability was maintained in periostin-HA ST2 cells in the 2.4 mM H_2_O_2_ treated condition (Fig. S3), suggesting that periostin gives cells tolerance against the stress, and inhibits cell death, similarly to *in vivo* data shown in Fig. 5.

### Concluding remarks

Recently, it was proposed that periostin influences Notch signaling by binding to an integrin receptor, which inhibits the expression of Dlk1 (negative regulator of Notch signaling) to enhance Notch signaling [27]. Different from this previous report, we propose that the Notch1 protein level is decreased under the oxidative stress condition but that periostin physically associates with the Notch1 precursor, and then maintains Notch1 protein expression and subsequent signaling under this stress condition. Furthermore, Notch signaling is well known to play an important role in anti-cell death function under mechanical stress [26], [28], which consistent with our results.

Our present results provide important insights into the regulation of Notch1 expression under the stress condition, and into periostin as a regulator of Notch1 expression, which is another unidentified function of periostin.

## Materials and Methods

### Animals

Periostin −/− mice were generated in our laboratory as described previously [9], [13].

All animal experiments were performed following The Regulation on animal experimentation of Tokyo Institute of Technology. This study was approved by the Animal Care and Use Committee of Tokyo Institute of Technology.

### Plasmid construction

The expression constructs, periostin/pCXN2, Notch1-FLAG/pCMV-Tag4 (Strategene), NdEGF-Myc/pSecTag2 (Invitrogen), and EGF8-32-Myc/pSecTag2 were previously described [2], [15], [29]. Periostin-Myc or periostin-HA was ligated into pCAGI-puro after having been fused to Myc or HA tag, respectively, by PCR methods. Periostin cDNA, which is inserted into pCXN2, was used as the template. Periostin-HA and AcGFP (Clontech) were also subcloned into pMXs-IRES-puro.

### Cell Cultures and transient transfection

ST2 cells and HEK293T cells were obtained from the Cell Bank of RIKEN BioResource Center. ST2 cells were cultured in α-MEM (Nacalai), and HEK293T cells and Plat-E retroviral packaging cells were cultured in low-glucose DMEM (Nacalai). One day before transfection, HEK293T cells were plated at 1.0×10^6^ cells/well in 6-well plates, and after culturing, 90% confluent cells were transfected with plasmids by using Lipofectamine 2000 (Invitrogen) according to the manufacturer's instructions. Each medium was supplemented with 10% FBS (MultiSer™; Thermo Trace), penicillin G (100 U/ml), and streptomycin (100 µg/ml). Cells were incubated at 37°C in a humidified atmosphere of 5% CO_2_ in air.

### Retroviral infection

Plat-E cells were transfected with Periostin-HA or AcGFP/pMxs-IRES-puro to prepare virus-containing supernatants. One day after ST2 cells (5.0×10^4^) were plated on a 6-well plate dish, the cells were retrovirally infected (MOI = 0.02). Following the infection, ST2 cells were selected by puromycin (Nacalai) to obtain stable infectants.

### Preparation of cells and molar PDL lysates

Two days after transfection, over-confluent HEK293T cells were lysed in lysis buffer [150 mM NaCl, 100 mM Tris-Cl (pH 7.4), 1% TritonX-100, 0.5% NP-40, 1 mM EDTA, 1 mM EGTA, 1 µg/ml leupeptin, 0.5 mM PMSF] for immunoprecipitation. For western blotting (Fig. 2A), PDL tissues were scraped from the mandibular molar of 12-week-mice, and were directly diluted into SDS sample buffer [50 mM Tris (pH 6.8), 2% SDS, 0.1% Bromophenol Blue, and 10% glycerol with 50 mM DTT]. For Fig. 3, proteins were extracted from the ST2 cells, according to the manufacturer's instructions using Isogen (Nippon Gene Co., LTD.). The mRNAs were extracted at the same time.

### Immunoprecipitation

Anti-FLAG antibody (rabbit; SIGMA) or anti-Myc antibody (A-14; Santa Cruz) were used for the precipitation. Whole-cell lysates or the IP samples were mixed with SDS sample buffer, and boiled for 5 min and used for Western blotting. For the primary antibody, we used anti-HA antibody (HA-7; SIGMA), anti-FLAG antibody (M2; SIGMA), anti-Myc antibody (9E10; Santa Cruz), anti-rodent Notch1 antibody (8G10; Santa Cruz) or anti-periostin antibody [2]. Signals were detected by using HRP-conjugated secondary antibodies.

### Western blot analysis

Samples, which were mildly sonicated and heated for 30 min at 70°C, were separated by SDS-PAGE and blotted onto nitrocellulose membranes. Subsequently, the membranes were blocked with TBS containing 5% skim milk and then incubated with primary antibody; anti-Notch1 antibody (M20; Santa Cruz), or anti-β-actin antibody (AC-15; SIGMA). Signals were detected by using HRP-conjugated secondary antibodies.

### Semi-quantitative RT-PCR analysis

Total RNA was extracted from the PDL tissues of a mandibular molar from a 12-week-old mouse or from ST2 cells by using Isogen according to the manufacturer's instructions. Each RNA sample (1 µg) was reverse-transcribed by using oligo-dT primer in a volume of 10 µl (Life Sciences, Inc.), adjusted to a final volume of 100 µl with TE buffer, and used for the RT-PCR analysis. RT-PCR analysis was performed by using r-Taq (TAKARA) according to the manufacturer's instructions. The sequences of the primers were as follow: for Notch1, 5′-GCAGCGTGTTAATGACTTCC-3′ and 5′-GGCATAGACAGCGGTAGAAAG-3′; for Hey1, 5′-TCCAAACTGTCTCCACCGC-3′ and 5′-AAGATTCCCCACGCTGAGG-3′; for Bcl-xL, 5′-TGGAGTAAACTGGGGGTCGCATCG-3′ and 5′-AGCCACCGTCATGCCCGTCAGG-3′; and for GAPDH, 5′-ACTTTGTCAAGCTCATTTCC-3′ and 5′-TGCAGCGAACTTTATTGATG-3′.

### Immunohistochemical analysis

After anesthesia with diethyl ether and pentobarbital (Nembutal, Dinabot, Osaka, Japan), wild-type and periostin−/− mice were fixed with 4% paraformaldehyde in 0.1 M cacodylate buffer (pH 7.4) by perfusion through the cardiac left ventricle. Mandibules from wild-type and periostin−/− mice were extracted, decalcified with 10% EDTA for 1 month and embedded in paraffin. Dewaxed paraffin sections were pretreated with 0.3% hydrogen peroxide for 20 min, and then with 1% bovine serum albumin (Serological Proteins Inc.) in PBS for 30 min. They were incubated with anti-Notch1 antibody (M-20; Santa Cruz) for 2 hr at room temperature. After having been rinsed with PBS, they were next incubated with horseradish peroxidase (HRP)-conjugated anti-goat Igs (Chemicon International Inc.) for 1 hr. Visualization by light microscopy was carried out by using diaminobentidzine as the substrate for the enzymic reaction with cell nuclei being counterstained with methyl green. Some specimens were examined under an immunofluorescence microscope. After incubation with the primary antibody against Notch1, the specimens were reacted with FITC-conjugated anti-goat Igs (Kirkegaard & Perry Laboratories Inc., Gaithersburg, MD). All sections were counterstained with DAPI prior to being observed under a Nikon Eclipse E800 microscope (Nikon Instruments Inc., Tokyo, Japan).

### Experimental model for tooth movement

The molars of 12-week-old mice were used for experiments. Elastic bands of 0.5 mm thickness were inserted between the first and second upper molars of the wild-type mice and littermates homozygous for periostin gene depletion and left there for 3 days. The first molars and peripheral alveolar bones were then extracted, fixed with 4% paraformaldehyde diluted in 0.1 M phosphate buffer (pH 7.4), and processed for light and electron microscopic observations. As described above, the specimens for light microscopic observations were decalcified with 10% EDTA and embedded in paraffin. For transmission electron microscopy, fixed specimens were decalcified with 4.13% EDTA, post-fixed with OsO_4_, dehydrated, and embedded in epoxy resin (Epon 812, Taab, Berkshire, UK). The distal roots and interradicular septum of the upper first molars were examined under a light microscope (Nikon Eclipse E800 microscope) and a transmission electron microscope (Hitachi H-7100, Hitachi Co., Tokyo, Japan).

## Supporting Information

Figure S1 Reduction in cell-surface Notch1 protein expression in periosteum of Pn−/− mice: (A) Immunofluorescence analysis of periosteum from 12-week-old wild-type mice and Pn−/− mice was performed with anti-C-terminal-Notch1 antibody (M20) (red). Cell nuclei were stained with DAPI. Arrowheads indicate the stain that would represent cell-surface Notch1. The fluorescence indicating cell-surface Notch1 was reduced in the Pn−/− periosteum compared with that in the wild-type one. Bars, 50 µm. (B) To confirm the localization of Notch1 in the periosteum of 12 week-old-mice, we co-localized Notch1 (red) with ALP (green), which is expressed on the surface of periosteal cells, indicating that Notch1 is localized there. The anti-ALP antibody used was previously described [1]. Bar, 50 µm. Reference: 1. Oda K, Amaya Y, Fukushi-Irie M, Kinameri Y, Ohsuye K, et al. (1999) A general method for rapid purification of soluble versions of glycosylphosphatidylinositol-anchored proteins expressed in insect cells: an application for human tissue-nonspecific alkaline phosphatase. J Biochem 126: 694–699.
(2.84 MB TIF)

Figure S2 Periostin regulates Bcl-xL expression through the maintenance of N1™ level: (A) Western blot analyses for N1™ and Bcl-xL were performed using anti-C-terminal-Notch1 antibody (M20) and anti-Bcl-xL antibody (54H6: Cell Signaling), respectively. Confluent periostin-HA or AcGFP ST2 cells were stressed with 2.4 mM H_2_O_2_ for 24 hours. The left 2 lanes show no stressed AcGFP or periostin-HA ST2 cells, and the right 2 lanes show 2.4 mM H_2_O_2_ stressed cells. In the periostin-HA cells, the N1™ level was maintained and showed 6 fold difference, and Bcl-xL expression was also maintained and showed 10 fold difference compared with those in the AcGFP cells, respectively, suggesting that periostin maintains S1-cleaved heterodimeric Notch1 and Bcl-xL expression in the H_2_O_2_ induced oxidative stress condition. β-actin was used for a loading control. Results indicate the relative density normalized to β-actin. (means±SEM, n = 3; *p<0.05.) (B) Western blot analysis for Bcl-xL. 2.4 mM H_2_O_2_ stressed confluent periostin-HA cells were treated with or without 10 µM γ-Secretase inhibitor XX (GSI; Calbiochem) for 24 hours. Bcl-xL expression in GSI treated cells was decreased and showed 1/2.5 difference compared with that of no treated cells, suggesting that periostin controls Bcl-xL expression through regulation of the N1™ level in the H_2_O_2_ induced oxidative stress condition. β-actin was used for a loading control. Results indicate the relative density normalized to β-actin. (means±SEM, n = 3; *p<0.05).
(0.33 MB TIF)

Figure S3 Periostin suppressed cell death in the H_2_O_2_ induced stress condition: The cell viability assay was performed by using Cell Count Reagent SF (nacalai), according to the manufacturer's instructions. Confluent periostin or AcGFP ST2 cells were stressed with 2.4 mM H_2_O_2_ for 24 hours. After that, we added Cell Count Reagent SF into the cells, and measured absorbance at 490 nm by Micro Plate Reader (Model 550, Bio-Rad). The cell viability was reduced in periostin cells, indicating that periostin suppressed cell death in the stress condition in vitro. Results indicate the absorbance. (meansÂ±SEM, n = 4; *p<0.05 compared with the AcGFP cells.)
(0.06 MB TIF)

## References

[1] Bornstein P, Sage EH (2002). Matricellular proteins: extracellular modulators of cell function.. Curr Opin Cell Biol.

[2] Horiuchi K, Amizuka N, Takeshita S, Takamatsu H, Katsuura M (1999). Identification and characterization of a novel protein, periostin, with restricted expression to periosteum and periodontal ligament and increased expression by transforming growth factor beta.. J Bone Miner Res.

[3] Takeshita S, Kikuno R, Tezuka K, Amann E (1993). Osteoblast-specific factor 2: cloning of a putative bone adhesion protein with homology with the insect protein fasciclin I.. Biochem J.

[4] Norris RA, Borg TK, Butcher JT, Baudino TA, Banerjee I (2008). Neonatal and adult cardiovascular pathophysiological remodeling and repair: developmental role of periostin.. Ann N Y Acad Sci.

[5] Kruzynska-Frejtag A, Machnicki M, Rogers R, Markwald RR, Conway SJ (2001). Periostin (an osteoblast-specific factor) is expressed within the embryonic mouse heart during valve formation.. Mech Dev.

[6] Goetsch SC, Hawke TJ, Gallardo TD, Richardson JA, Garry DJ (2003). Transcriptional profiling and regulation of the extracellular matrix during muscle regeneration.. Physiol Genomics.

[7] Lindner V, Wang Q, Conley BA, Friesel RE, Vary CP (2005). Vascular injury induces expression of periostin: implications for vascular cell differentiation and migration.. Arterioscler Thromb Vasc Biol.

[8] Oka T, Xu J, Kaiser RA, Melendez J, Hambleton M (2007). Genetic manipulation of periostin expression reveals a role in cardiac hypertrophy and ventricular remodeling.. Circ Res.

[9] Shimazaki M, Nakamura K, Kii I, Kashima T, Amizuka N (2008). Periostin is essential for cardiac healing after acute myocardial infarction.. J Exp Med.

[10] Rios HF, Ma D, Xie Y, Giannobile WV, Bonewald LF (2008). Periostin is essential for the integrity and function of the periodontal ligament during occlusal loading in mice.. J Periodontol.

[11] Norris RA, Damon B, Mironov V, Kasyanov V, Ramamurthi A (2007). Periostin regulates collagen fibrillogenesis and the biomechanical properties of connective tissues.. J Cell Biochem.

[12] Gillan L, Matei D, Fishman DA, Gerbin CS, Karlan BY (2002). Periostin secreted by epithelial ovarian carcinoma is a ligand for alpha(V)beta(3) and alpha(V)beta(5) integrins and promotes cell motility.. Cancer Res.

[13] Kii I, Amizuka N, Minqi L, Kitajima S, Saga Y (2006). Periostin is an extracellular matrix protein required for eruption of incisors in mice.. Biochem Biophys Res Commun.

[14] Kessler CB, Delany AM (2007). Increased Notch 1 expression and attenuated stimulatory G protein coupling to adenylyl cyclase in osteonectin-null osteoblasts.. Endocrinology.

[15] Sakamoto K, Yamaguchi S, Ando R, Miyawaki A, Kabasawa Y (2002). The nephroblastoma overexpressed gene (NOV/ccn3) protein associates with Notch1 extracellular domain and inhibits myoblast differentiation via Notch signaling pathway.. J Biol Chem.

[16] Iso T, Kedes L, Hamamori Y (2003). HES and HERP families: multiple effectors of the Notch signaling pathway.. J Cell Physiol.

[17] Blaumueller CM, Qi H, Zagouras P, Artavanis-Tsakonas S (1997). Intracellular cleavage of Notch leads to a heterodimeric receptor on the plasma membrane.. Cell.

[18] Logeat F, Bessia C, Brou C, LeBail O, Jarriault S (1998). The Notch1 receptor is cleaved constitutively by a furin-like convertase.. Proc Natl Acad Sci U S A.

[19] Fiuza UM, Arias AM (2007). Cell and molecular biology of Notch.. J Endocrinol.

[20] Hecht JT, Sage EH (2006). Retention of the matricellular protein SPARC in the endoplasmic reticulum of chondrocytes from patients with pseudoachondroplasia.. J Histochem Cytochem.

[21] Kim BY, Olzmann JA, Choi SI, Ahn SY, Kim TI (2009). Corneal dystrophy-associated R124H mutation disrupts TGFBI interaction with Periostin and causes mislocalization to the lysosome.. J Biol Chem.

[22] Paravicini TM, Touyz RM (2006). Redox signaling in hypertension.. Cardiovasc Res.

[23] van der Vlies D, Pap EH, Post JA, Celis JE, Wirtz KW (2002). Endoplasmic reticulum resident proteins of normal human dermal fibroblasts are the major targets for oxidative stress induced by hydrogen peroxide.. Biochem J.

[24] van der Vlies D, Makkinje M, Jansens A, Braakman I, Verkleij AJ (2003). Oxidation of ER resident proteins upon oxidative stress: effects of altering cellular redox/antioxidant status and implications for protein maturation.. Antioxid Redox Signal.

[25] Karlstrom H, Beatus P, Dannaeus K, Chapman G, Lendahl U (2002). A CADASIL-mutated Notch 3 receptor exhibits impaired intracellular trafficking and maturation but normal ligand-induced signaling.. Proc Natl Acad Sci U S A.

[26] Morrow D, Sweeney C, Birney YA, Cummins PM, Walls D (2005). Cyclic strain inhibits Notch receptor signaling in vascular smooth muscle cells in vitro.. Circ Res.

[27] Tkatchenko TV, Moreno-Rodriguez RA, Conway SJ, Molkentin JD, Markwald RR (2009). Lack of periostin leads to suppression of Notch1 signaling and calcific aortic valve disease.. Physiol Genomics.

[28] Gude NA, Emmanuel G, Wu W, Cottage CT, Fischer K (2008). Activation of Notch-mediated protective signaling in the myocardium.. Circ Res.

[29] Sakamoto K, Chao WS, Katsube K, Yamaguchi A (2005). Distinct roles of EGF repeats for the Notch signaling system.. Exp Cell Res.

