# Supplementary figures and images for: Periostin Associates with Notch1 Precursor to Maintain Notch1 Expression under a Stress Condition in Mouse Cells
Source: PLoS One. 2010 Aug 18;5(8):e12234. doi: 10.1371/journal.pone.0012234 (PMC2923609; doi:10.1371/journal.pone.0012234)

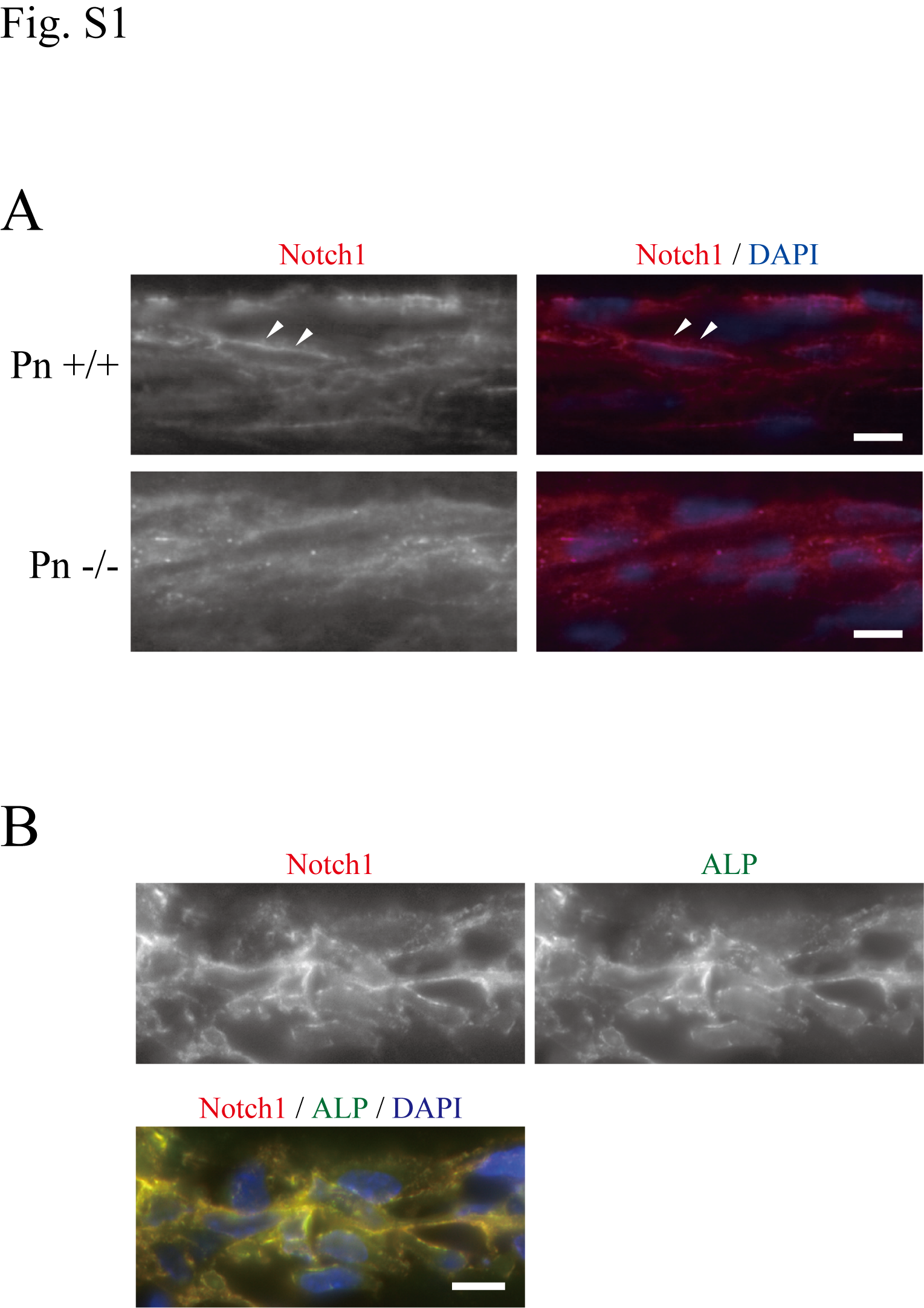

Supplement: Figure S1 — Reduction in cell-surface Notch1 protein expression in periosteum of Pn−/− mice: (A) Immunofluorescence analysis of periosteum from 12-week-old wild-type mice and Pn−/− mice was performed with anti-C-terminal-Notch1 antibody (M20) (red). Cell nuclei were stained with DAPI. Arrowheads indicate the stain that would represent cell-surface Notch1. The fluorescence indicating cell-surface Notch1 was reduced in the Pn−/− periosteum compared with that in the wild-type one. Bars, 50 µm. (B) To confirm the localization of Notch1 in the periosteum of 12 week-old-mice, we co-localized Notch1 (red) with ALP (green), which is expressed on the surface of periosteal cells, indicating that Notch1 is localized there. The anti-ALP antibody used was previously described [1]. Bar, 50 µm. Reference: 1. Oda K, Amaya Y, Fukushi-Irie M, Kinameri Y, Ohsuye K, et al. (1999) A general method for rapid purification of soluble versions of glycosylphosphatidylinositol-anchored proteins expressed in insect cells: an application for human tissue-nonspecific alkaline phosphatase. J Biochem 126: 694–699. (2.84 MB TIF) [file pone.0012234.s001.tif]

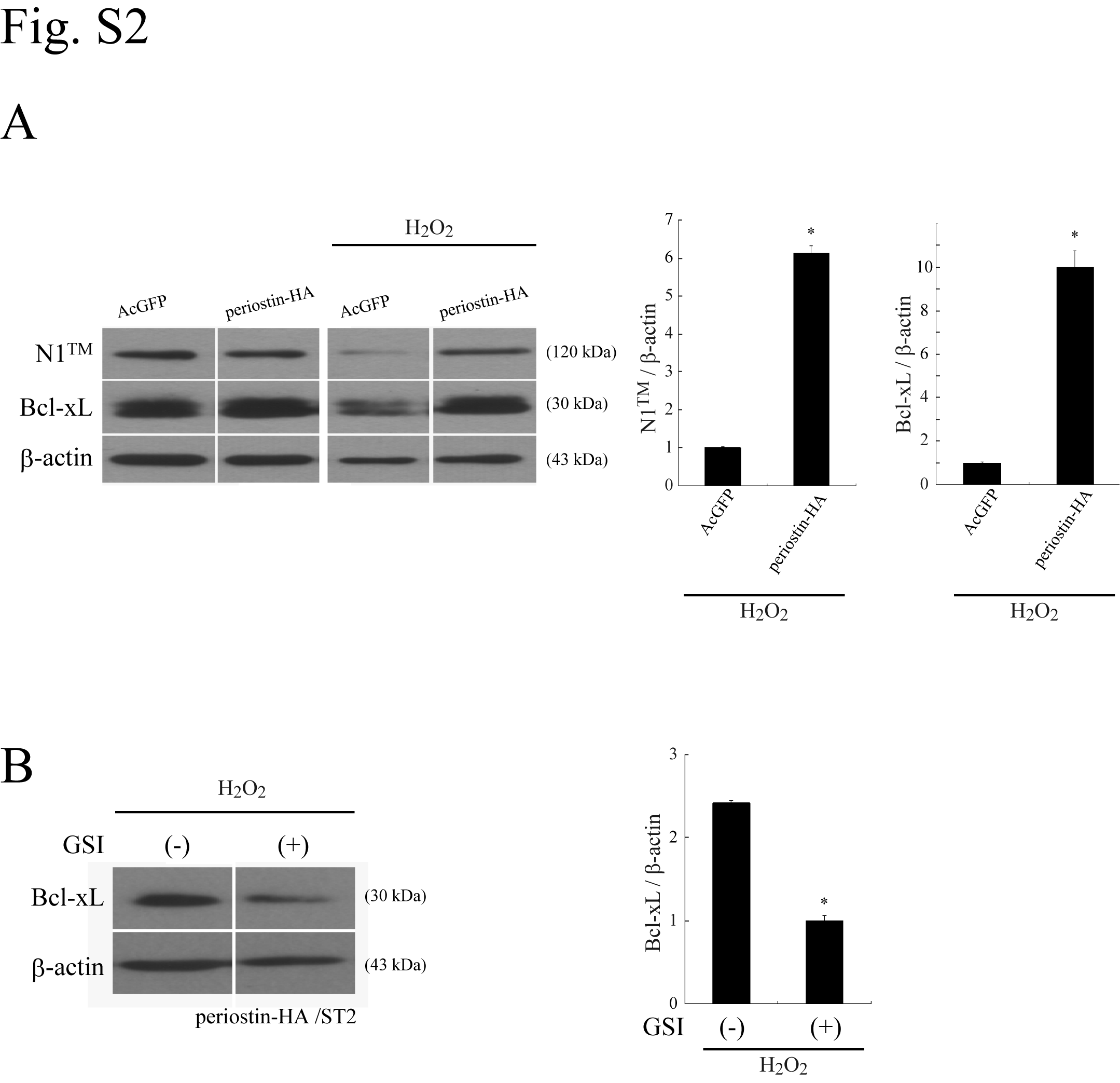

Supplement: Figure S2 — Periostin regulates Bcl-xL expression through the maintenance of N1™ level: (A) Western blot analyses for N1™ and Bcl-xL were performed using anti-C-terminal-Notch1 antibody (M20) and anti-Bcl-xL antibody (54H6: Cell Signaling), respectively. Confluent periostin-HA or AcGFP ST2 cells were stressed with 2.4 mM H2O2 for 24 hours. The left 2 lanes show no stressed AcGFP or periostin-HA ST2 cells, and the right 2 lanes show 2.4 mM H2O2 stressed cells. In the periostin-HA cells, the N1™ level was maintained and showed 6 fold difference, and Bcl-xL expression was also maintained and showed 10 fold difference compared with those in the AcGFP cells, respectively, suggesting that periostin maintains S1-cleaved heterodimeric Notch1 and Bcl-xL expression in the H2O2 induced oxidative stress condition. β-actin was used for a loading control. Results indicate the relative density normalized to β-actin. (means±SEM, n = 3; *p<0.05.) (B) Western blot analysis for Bcl-xL. 2.4 mM H2O2 stressed confluent periostin-HA cells were treated with or without 10 µM γ-Secretase inhibitor XX (GSI; Calbiochem) for 24 hours. Bcl-xL expression in GSI treated cells was decreased and showed 1/2.5 difference compared with that of no treated cells, suggesting that periostin controls Bcl-xL expression through regulation of the N1™ level in the H2O2 induced oxidative stress condition. β-actin was used for a loading control. Results indicate the relative density normalized to β-actin. (means±SEM, n = 3; *p<0.05). (0.33 MB TIF) [file pone.0012234.s002.tif]

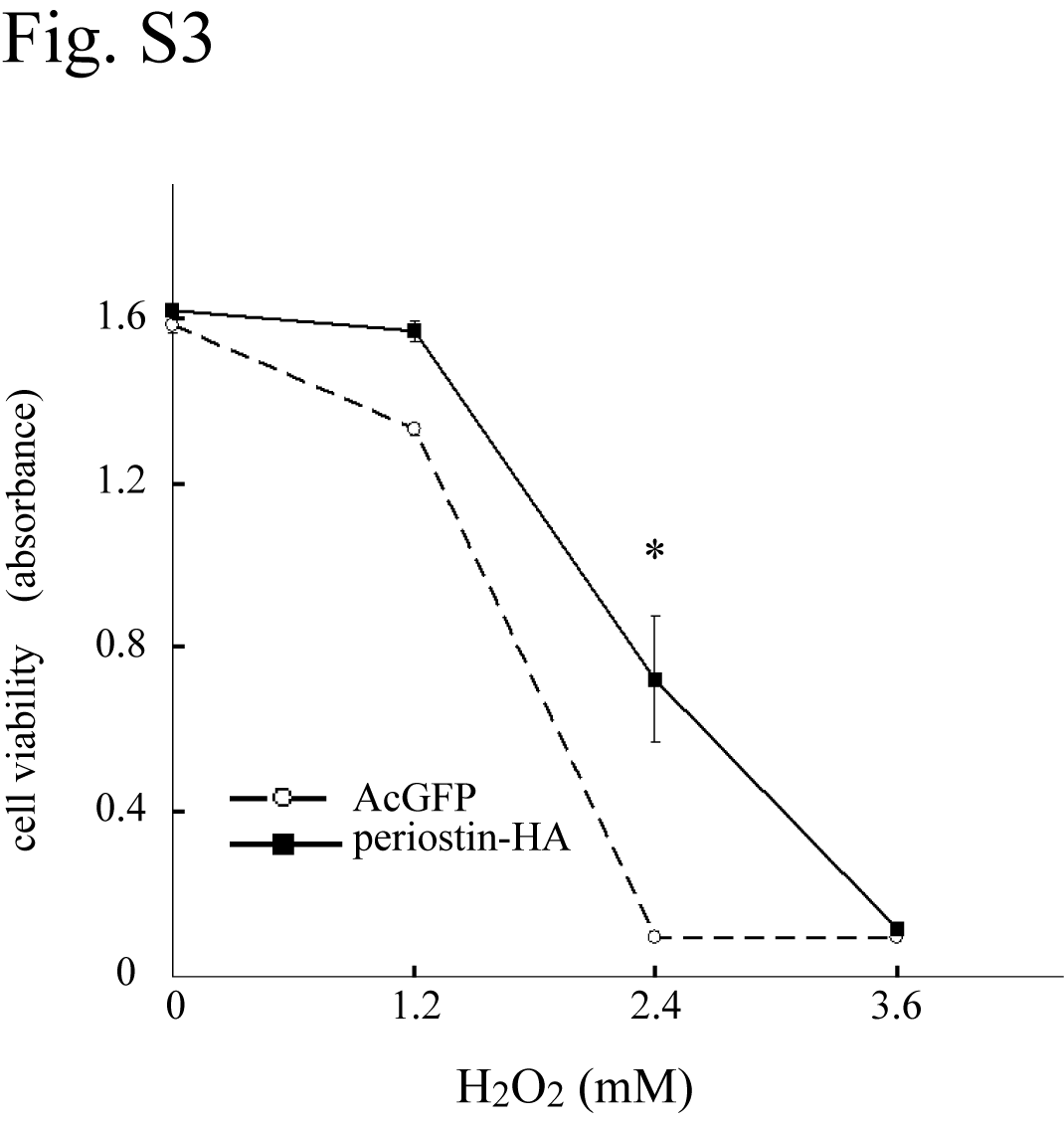

Supplement: Figure S3 — Periostin suppressed cell death in the H2O2 induced stress condition: The cell viability assay was performed by using Cell Count Reagent SF (nacalai), according to the manufacturer's instructions. Confluent periostin or AcGFP ST2 cells were stressed with 2.4 mM H2O2 for 24 hours. After that, we added Cell Count Reagent SF into the cells, and measured absorbance at 490 nm by Micro Plate Reader (Model 550, Bio-Rad). The cell viability was reduced in periostin cells, indicating that periostin suppressed cell death in the stress condition in vitro. Results indicate the absorbance. (meansÂ±SEM, n = 4; *p<0.05 compared with the AcGFP cells.) (0.06 MB TIF) [file pone.0012234.s003.tif]
